# Rediscovery of Red Wolf Ghost Alleles in a Canid Population Along the American Gulf Coast

**DOI:** 10.3390/genes9120618

**Published:** 2018-12-10

**Authors:** Elizabeth Heppenheimer, Kristin E. Brzeski, Ron Wooten, William Waddell, Linda Y. Rutledge, Michael J. Chamberlain, Daniel R. Stahler, Joseph W. Hinton, Bridgett M. vonHoldt

**Affiliations:** 1 Ecology and Evolutionary Biology, Princeton University, Princeton, NJ 08544, USA; eh7@princeton.edu; 2 School of Forest Resources & Environmental Science, Michigan Technological University, Houghton, MI 49931, USA; 3 Wildlife Biologist, Galveston, TX 77550, USA; Ronald.B.Wooten@usace.army.mil; 4 Zoological and Environmental Education Department, Point Defiance Zoo & Aquarium, Tacoma, WA 98407, USA; firthoclyde53@gmail.com; 5 Biology Department, Trent University, Peterborough, ON K9L 1Z8, Canada; lrutledge@trentu.ca; 6 Warnell School of Forestry and Natural Resources, University of Georgia, Athens, GA 30602, USA; mchamb@uga.edu (M.J.C.); jhinton@uga.edu (J.W.H.); 7 Yellowstone Center for Resources, National Park Service, Yellowstone National Park, WY 82190, USA; dan_stahler@nps.gov

**Keywords:** allele sharing, coyotes, ghost alleles, RADseq, red wolves, remnant genomes

## Abstract

Rediscovering species once thought to be extinct or on the edge of extinction is rare. Red wolves have been extinct along the American Gulf Coast since 1980, with their last populations found in coastal Louisiana and Texas. We report the rediscovery of red wolf ghost alleles in a canid population on Galveston Island, Texas. We analyzed over 7000 single nucleotide polymorphisms (SNPs) in 60 canid representatives from all legally recognized North American *Canis* species and two phenotypically ambiguous canids from Galveston Island. We found notably high Bayesian cluster assignments of the Galveston canids to captive red wolves with extensive sharing of red wolf private alleles. Today, the only known extant wild red wolves persist in a reintroduced population in North Carolina, which is dwindling amongst political and taxonomic controversy. Our rediscovery of red wolf ancestry after almost 40 years introduces both positive opportunities for additional conservation action and difficult policy challenges.

## 1. Introduction

Red wolves (*Canis rufus*) once inhabited the southeastern United States but were declared extinct in the wild by 1980 due to habitat loss, predator control programs, disease, and interbreeding with encroaching coyotes (*Canis latrans*) [1]. In 1967, the U.S. Fish and Wildlife Service (USFWS) listed red wolves as endangered under the U.S. Endangered Species Preservation Act due to their rapid population decline in the American south, and subsequently, red wolves were among the first species listed on the 1973 Endangered Species Act (ESA), the Unites States’ landmark environmental law [1]. With red wolves on the brink of extinction, recovery was initiated through trapping what were believed to be the last wild red wolves along the Gulf Coast of Louisiana and Texas in the 1970s [1,2,3,4,5]. Individuals were selected as founders for the captive breeding program based on morphology and behavioral traits considered to be species informative [6,7]. Over 240 canids were trapped from coastal Louisiana and Texas between 1973 and 1977 [6]. Forty individuals were selected for captive breeding, of which 17 were deemed 100% wolf. However, only 14 wolves successfully reproduced and became the founders from which all red wolves in the recovery program descend.

Due to the successful captive breeding program, red wolves were restored to the landscape in North Carolina less than a decade after being declared extinct in the wild [6]. This historic event represented the first attempt to reintroduce a wild–extinct species in the United States and set a precedent for returning wild–extinct wildlife to the landscape. The success of the red wolf recovery program was the foundation upon which other wolf introductions were guided, including the gray wolf (*C. lupus*) reintroduction to the northern Rocky Mountains in Yellowstone National Park, Wyoming, and central Idaho, and the ongoing restoration efforts for the Mexican wolves (*C. lupus baileyi*) in the southwest [8,9]. Although successful by many measures [7], the North Carolina experimental population (NCEP) of red wolves was reduced by the USFWS in response to negative political pressure from the North Carolina Wildlife Resource Commission and a minority of private landowners [10]. Further, gunshot-related mortalities have increased the probability that wolf packs deteriorate before the breeding season, which facilitates the establishment of coyote–wolf breeding pairs [11,12]. Consequently, the NCEP has fewer than 40 surviving members [13] and red wolves are once again on the brink of extinction in the wild.

Interbreeding between red wolves and coyotes is well documented and is viewed as a threat to red wolf recovery [14]. When historic populations of red wolves along the Gulf Coast were surveyed, it was feared that these coastal populations were the last remnants of pre-recovery wild wolves and were likely to quickly become genetically extinct through introgressive swamping of coyote genetics [15]. Yet, there continued to be reports of red wolves in rural regions of coastal Louisiana and Texas since the 1970s [5,16]. Previous efforts to detect surviving red wolves or their hybrids in the region proved unsuccessful [17]. However, the possibility remains that individuals with substantial red wolf ancestry have naturally persisted in isolated areas of the Gulf Coast. For example, body measurements of coyote-like canids in southwestern Louisiana were similar to those of confirmed red wolf–coyote hybrids in the NCEP [18,19]. These individuals would harbor ghost alleles of the original red wolves, with these alleles lost in the contemporary red wolf population during the extreme population bottleneck, drift, and inbreeding.

**Figure 1:**
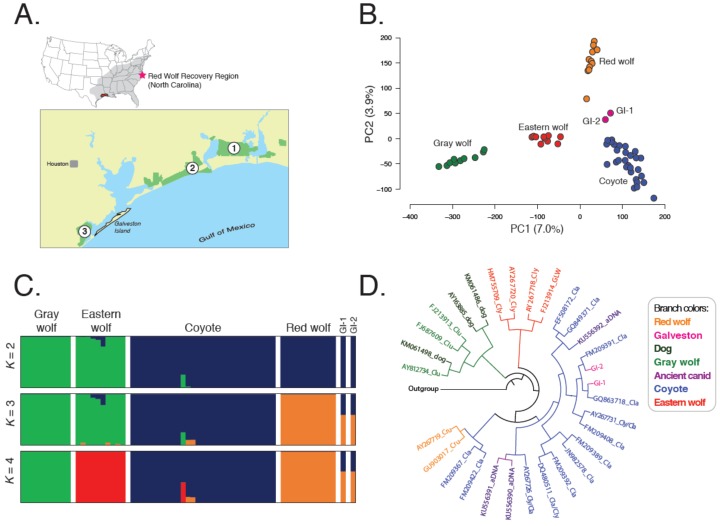
Analyses of genome-wide single nucleotide polymorphism (SNP) and mitochondrial DNA (mtDNA) data across all legally recognized wild *Canis* species and two canids from Galveston Island. (**A**) Map of area. Site 1 is Sabine National Wildlife Refuge, site 2 is McFadden National Wildlife Refuge, and site 3 is Brazoria National Wildlife Refuge. Cluster patterns were assessed across 7068 SNPs with a (**B**) principal component analysis (PCA; PC: principal component) and (**C**) admixture analysis of *K* = 2–4 partitions. (**D**) Clade membership was determined by reconstruction of the Bayesian haplotype tree with the highest posterior probability (Prob = 0.98) from 234 bp of mtDNA sequence data from the control region; taxonomic designation of eastern wolf is based on assigned clade and sample location, not necessarily field identification.

**Figure 2:**
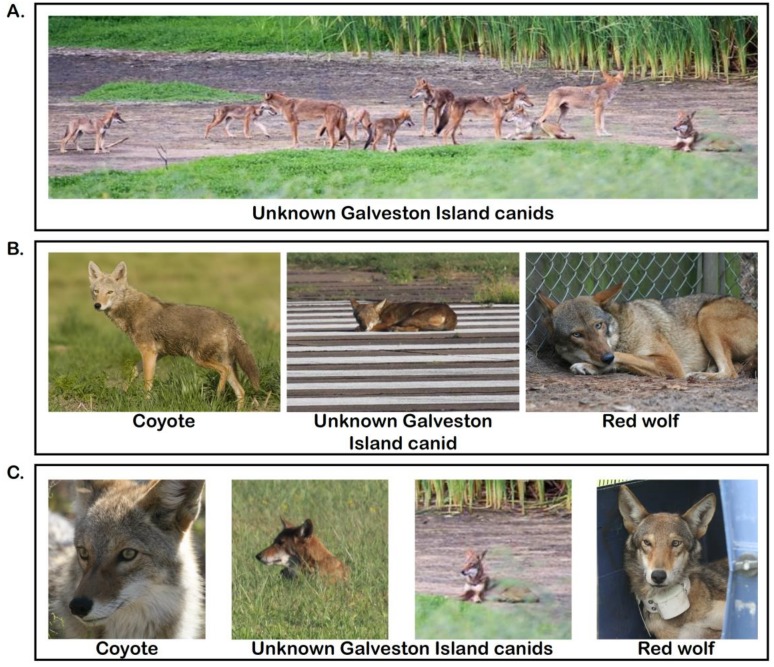
Photographic comparison of coyotes, Galveston Island (GI) canids, and red wolves. Photo credit and location as follows: (**A**) Pack of GI canids, Galveston Island, TX, credit: R. Wooten. (**B**) Western coyote, Intermountain West, United States, credit: Wikimedia commons, Rich Keen/DPRA. GI canid laying on airport runway, Galveston Island, TX, credit: R. Wooten. Captive female red wolf, Alligator River National Wildlife Refuge, NC, credit: R. Nordsven, USFWS. (**C**) Western coyote, Joshua Tree National Park, CA, credit: Wikimedia commons, Michael Vamstad/NPS. Headshots of GI canids, Galveston Island, TX, credit: R. Wooten. Wild juvenile male red wolf prior to release, Albemarle Peninsula, NC, credit R. Nordsven, USFWS.

For red wolf ghost alleles to persist, a remnant Gulf Coast population would need to be relatively isolated from frequent interbreeding with coyotes [14]. Although red wolves that co-occur with coyotes in the NCEP exhibit assortative mating patterns [20], a geographic island would promote genetic isolation and the persistence of red wolf alleles. We report evidence that Galveston Island, Texas (TX) may represent one such location. All contemporary red wolves descended from individuals trapped from Jefferson, Chambers, southern Orange, and eastern Galveston counties in Texas and Cameron and southern Calcasieu parishes in Louisiana [16] (Figure 1). Given Galveston Island’s location and isolation from the mainland, it is a probable region to harbor red wolf ghost alleles. Recent images captured of Galveston Island canids (Figure 2) piqued interest of local naturalists and two genetic samples were taken from roadkill individuals. Accordingly, our objective was to conduct genomic analyses and determine if there was evidence of red wolf ancestry in modern-day Galveston Island canids.

## 2. Materials and Methods

### 2.1. DNA Sequencing and Bioinformatic Processing

We obtained tissue samples from two roadkill canids of unknown taxonomic affiliation on Galveston Island (GI), TX and extracted genomic DNA using the Qiagen DNeasy blood and tissue kit (Qiagen, Maryland, USA), following the manufacturer’s instructions. For comparison, we selected reference samples that represented all wild canid evolutionary lineages in North America that could have contributed to ancestry and genetic variation of the two GI canids: 29 coyotes from Alabama, Louisiana, Oklahoma, and Texas; 10 gray wolves from Yellowstone National Park; 10 eastern wolves (*C. lycaon*) from Algonquin Provincial Park in Ontario; and 11 red wolves from the Special Survival Plan captive breeding program that collectively represent the 12 founders genetically represented in extant red wolves (Table S1). All samples were collected under Princeton Institutional Animal Care and Use Committee (IACUC) #1961A-13.

We estimated variation across the genome of all 62 canids using a modified RADseq protocol [21]. We digested DNA with *Sbf1* (New England Biolabs, Ipswich, MA, USA), ligated a unique barcode adapter to the fragments, and pooled between 96 and 153 samples. Each pool was subsequently sheared to 400 bp in a Covaris LE220 at Princeton University’s Lewis Siegler Institute Genomics Core Facility. We recovered ligated fragments using a streptavidin bead binding assay (Dynabeads M-280, Thermo Fisher Scientific, Waltham, MA, USA) and prepared genomic libraries for Illumina HiSeq sequencing following either the standard TruSeq protocol for the NEBNext Ultra or NEBNext UltraII DNA Library Prep Kit (New England Biolabs). We conducted a size selection step using Agencourt AMPure XP beads (Beckman Coulter, Brea, CA, USA) to retain fragments 300–400 bp in size. We also used AMPure XP beads for library purification. We standardized genomic libraries to 10nM for 2 × 150 nt sequencing on an Illumina HiSeq 2500 platform.

Prior to variant calling, we filtered raw sequence data to retain reads that contained a barcode and the restriction enzyme cut-site using a custom perl script (flip_trim_sbfI_170601.pl, Supporting Information). We then discovered variant sites following the recommended STACKS v1.42 pipeline for reference mapped data [22]. Reads were demultiplexed using *Process_Radtags*, allowing a mismatch of two to rescue barcodes. We discarded reads with an uncalled base or with an average quality score (≤10) within a sliding window equivalent to 15% of the total read length. We removed PCR duplicates using *Clone_Filter* with default parameters. All samples were mapped to the Canfam3.1 assembly of dog genome [23] with STAMPY v 1.0.31 [24]. We filtered mapped reads in Samtools v 0.1.18 [25] to retain those with MAPQ > 96 and exported as a bam file. Variant calling was then completed in STACKS. We required a minimum stack depth of 3 reads (-m) in *pstacks* and allowed a maximum per locus missingness of 10% in *populations*. Further, to reduce biases resulting from linked markers, we enabled the—*write_single_snp* flag in *populations* and filtered for statistical linkage disequilibrium (LD) across sites using the—*indep-pairwise* 50 5 0.5 flag in Plink v1.90b3i [26]. We conducted a final filtering to retain sites that also had a minimum minor allele of 1%. Demultiplexed and clone-filtered sequencing reads, along with the associated bam files, have been submitted to the NCBI sequence read archive (SRA) under accession number: PRJNA507274.

We calculated the standard metrics of genomic diversity (observed heterozygosity, *H*_O_; private allele count, *N*_PA,_ Pairwise F_ST_) in STACKS and evaluated the significance of pairwise estimates using a pairwise Wilcoxon rank sum test implemented in *R* with a false discovery rate correction for multiple testing (FDR < 0.05). Allelic richness (A_r_) and private allelic richness (PA_r_) were calculated using rarefaction in ADZE [27] with a maximum tolerance of 10% missing data.

### 2.2. Population Structure Analyses

We evaluated genetic clusters with a principal component analysis (PCA) in *flashPCA* [28]. Additional PCAs were conducted on a subset of samples for specific comparisons (i.e., only reference coyotes, red wolves, and the GI individuals). We implemented a maximum-likelihood analysis to infer population structure using the program ADMIXTURE v1.3. [29]. We evaluated between 1 and 10 genetic partitions (*K*), evaluated the fit of each partition using the cross-validation flag, and considered the best fit number of partitions to have the lowest cross-validation score. We first considered the entire dataset, with subsequent analyses run with only reference coyotes, red wolves, and GI canids.

Although this maximum-likelihood cluster analysis is useful for evaluating specific levels of data partitioning, it is not an explicit ancestry analysis. Using a Bayesian framework, we conducted a posterior probability assignment test in Structure v.2.3.4 [30] trained on two genetic reference groups (coyotes and red wolf) to obtain assignment proportions for each GI canid using 10,000 repetitions following a burn-in of 2500.

### 2.3. Private Allele Sharing Analyses

We explored the degree to which GI canids shared private alleles with reference groups to infer source population or recent introgression [27] based on the presence of excess private allele sharing—*indep-pairwise* [31]. To avoid spurious identification of private alleles due to missing data, we restricted analyses to loci that were 100% genotyped in each GI canid, considered each GI canid separately, and identified alleles private to each reference group in STACKS. We then determined the number of private alleles that were shared with the GI canids. We calculated shared private allelic richness with each reference group using a rarefaction approach in ADZE with a tolerance of 15% missing data. We estimated the frequency of each shared private allele in the corresponding reference coyote or red wolf population. This frequency distribution was binned as follows: The number of shared private alleles for each GI canid was divided by the total number of private alleles and binned in 10% frequency intervals based on the allele’s frequency in a corresponding reference population. From the genomic coordinates, we annotated each site as intergenic, intronic or exonic, or as a putative promoter (within 2 kb of a transcription start site) using a custom python script and the Ensembl gene database (chr_site.py; Supporting Information) [32]. We repeated this analysis after removing the LD filter and recalculated shared private alleles with the red wolf reference group. To identify unique genomic diversity, absent from reference groups, we evaluated alleles found only in GI-1 or GI-2. We calculated the identity-by-state between the two GI canids in Plink using the —bs-matrix argument.

### 2.4. Mitochondrial DNA Sequence Analysis

To investigate the matrilineal history of GI canids, we sequenced the mitochondrial DNA (mtDNA) control region that contains diagnostic haplotypes for both red wolves and ancient canids of the American southeast [33,34]. We amplified DNA using primers for the control region (Thr-L 15926: 5′-CAATTCCCCGGTCTTGTAAACC-3′; DL-H 16340: 5′ CCTGAAGTAGGAACCAGATG-3) and thermocycling conditions following Vilà et al. [35]. Amplified products were bidirectionally sequenced using a service provided by GeneWiz (New Jersey), where each sample was sequenced in duplicate to confirm ambiguous sites. Sequences were viewed, corrected, and aligned with Geneious v6.16 software [36]. We then compared a 234 bp consensus sequence from each GI canid to references on GenBank that represented all possible *Canis* ancestor lineages (Table S2). We estimated gene trees using Bayesian methods implemented in BEAST v1.8.4 [37], with a constant size coalescent tree prior, an uncorrelated lognormal relaxed molecular clock, and a random starting tree. We conducted two independent Markov Chain Monte Carlo (MCMC) analyses for 25 million steps, sampling every 2500 steps, and combined tree estimates from each run with LogCombiner v1.8.4 with a 10% burn-in. Convergence on the posterior distribution was determined based on viewing the log files in Tracerv1.6. To visualize the gene trees, we calculated the maximum clade credibility in TreeAnnotator v1.8.4 and uploaded the most likely tree in the Interactive Tree of Life v3.6.3 online platform [38].

## 3. Results

### 3.1. Galveston Island Canids Carry Red Wolf Genetic Signatures

We collected genomic and mtDNA sequences for two canids inhabiting Galveston Island, Texas of unknown taxonomic origin (Figure 2) and 60 reference North American canids and discovered 7068 genome-wide SNPs. Coyotes exhibited the highest genomic diversity and red wolves the lowest, with all pairwise comparisons significantly different (H_E_: Coyotes = 0.101, red wolf = 0.061) (Table 1 and Table S1). Red wolves were most differentiated from gray wolves (F_ST_ = 0.136) and most similar to coyotes (F_ST_: Red wolf–coyote = 0.040, red wolf–eastern wolf = 0.093, gray wolf–eastern wolf = 0.086, coyote–gray wolf = 0.062, coyote–eastern wolf = 0.042). A principal component analysis (PCA) revealed that clusters were concordant with taxonomic classifications (Figure 1A), consistent with previous analyses (vonHoldt et al., 2011), and spatial clustering of the two GI canids proximal to coyotes. When we restricted our analysis to only reference red wolves and coyotes, we observed a similar intermediate placement of the two GI canids (Figure S1A).

We used the maximum likelihood framework in ADMIXTURE (Alexander et al., 2009) to assess genetic structure and found the greatest support for three genetic groups (cv = 0.35) composed of gray and eastern wolves, coyotes, and red wolves, respectively (Figure 1C and Figure S1B). GI canids exhibited partial memberships only to red wolf and coyote groups (GI-1: *Q*_Red Wolf_ = 0.60, *Q*_Coyote_ = 0.40; GI-2: *Q*_Red Wolf_ = 0.60, *Q*_Coyote_ = 0.40). Strikingly, two Louisiana coyotes also exhibited nontrivial assignment to the red wolf genetic group (*Q*_Red Wolf_: LA-2 = 0.10; LA-3 = 0.11). A reduced analysis of only reference coyotes and red wolves revealed support for two genetic groups (coyotes and red wolves) (Figure S1B), with each GI canid displaying ~30% assignments to the red wolf cluster (Figure S1C). Further, *K* = 3 revealed distinct coyote subgroups corresponding to their historical range of Oklahoma and Texas, and their southeastern expansion across Louisiana and Alabama. GI canids retained nontrivial assignments to red wolves (*Q*_Red Wolf_: GI-1 = 0.27, GI-2 = 0.21) (Figure S2). Interestingly, the coyote proportions of the GI canids was attributed to the southeastern population (Q_Southeast_: GI-1 = 0.73; GI-2 = 0.79), consistent with interbreeding between red wolves and expanding coyote populations in the late 1970s. A posterior probability assignment test in STRUCTURE [30] revealed that each GI canid was explicitly assigned to one or more of the coyote and red wolf reference groups (QRed Wolf: GI-1 = 0.33, GI-2 = 0.28) (Figure S1C).

### 3.2. Galveston Canids Carry Red Wolf Private Alleles

We surveyed 6859 loci with nonmissing data for GI-1 and found that the most private alleles were shared with coyotes (*S*_PA_ = 184; *S*_PAr_ = 0.0102), followed by red wolves (*S*_PA_ = 21; *S*_PAr_ = 0.0059), a trend also supported after adjusting for unequal sampling (Table 2). We then surveyed 6391 loci for GI-2 and found similar trends of the greatest private allele sharing observed with coyotes (*S*_PA_ = 138; *S*_PAr_ = 0.0093) and red wolves (*S*_PA_ = 14; *S*_PAr_ = 0.0063) (Table 2).

**Figure 3:**
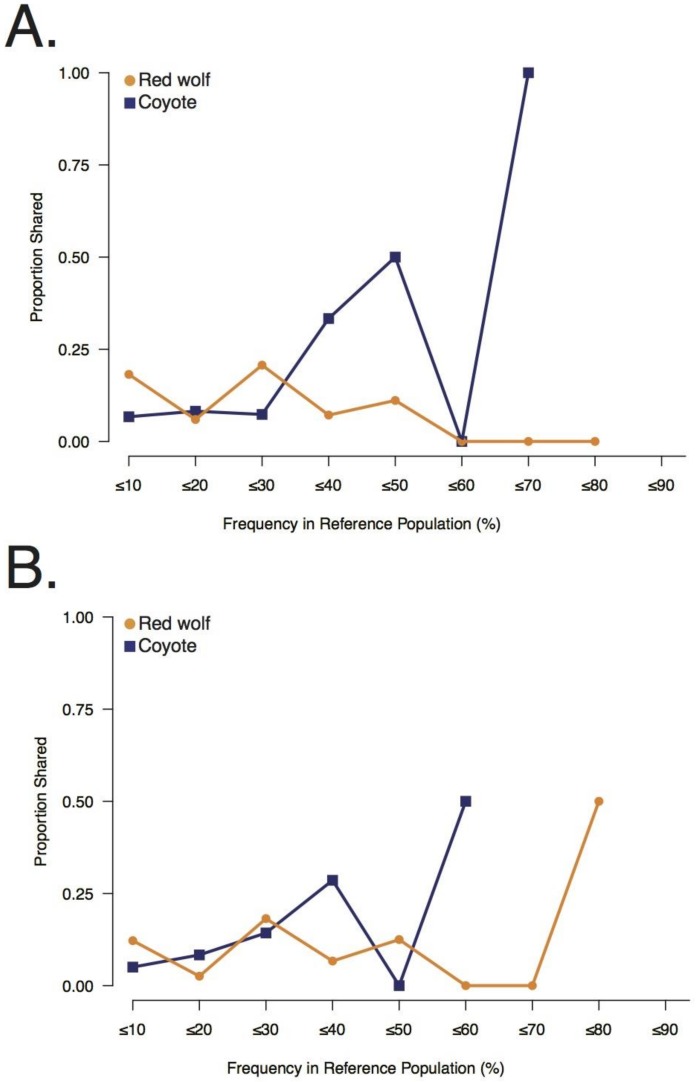
Sharing of red wolf and coyote private alleles with the two Galveston Island, Texas canids, (**A**) GI-1 and (**B**) GI-2, and their frequencies in their respective reference populations.

Collectively, the GI canids predominantly shared private alleles that were at low or moderate frequencies in the red wolf reference population (e.g., ≤10% propS: GI-1 = 0.18, GI-2 = 0.12) (Figure 3). Though GI-2 was observed to share some common red wolf private alleles as well (≤80%; propS = 0.50), shared common red wolf private alleles were not observed for GI-1 (>50%; propS = 0.00) (Figure 3). By contrast, each GI canid shared fewer low frequency coyote private alleles (e.g., ≤10%; propS: GI-1 = 0.07; GI-2 = 0.05) (Figure 3) and primarily shared common coyote alleles (e.g., ≤70%; propS: GI-1 = 1.00; ≤60% GI-2 = 0.50) (Figure 3). The shared private red wolf alleles found in the GI canid genomes were predominantly intergenic (*n*_intergenic_ = 19; *n*_exon_ = 3; *n*_intron_ = 7; *n*_promoter_ = 1) and in the heterozygous state (*H*_O_, GI-1: 0.76; GI-2: 0.71) (Table S3). We observed five overlapping shared red wolf private alleles between the two GI individuals; however, we estimated a high level of genome-wide allele sharing (identity-by-state = 0.93). When the LD filter was removed, we retained 8167 and 7609 SNPs in GI-1 and GI-2, respectively. GI-1 carried a total of 30 red wolf private alleles (*n*_homozygous_ = 8) and GI-2 carried 26 (*n*_homozygous_ = 8) (Figure S3). Although this provided a genome-level perspective of red wolf allele sharing, the resolution was not sufficient to conclusively identify contiguous shared private alleles in extended linkage disequilibrium due to recent admixture.

The GI canids carried 21 alleles that were absent from all reference populations, distributed throughout their genomes (intergenic = 16; exon = 1; intron = 4) (Figure S3, Table S4B). GI-1 carried 14 private alleles and was homozygous for 50% of loci, GI-2 carried 16 private alleles and was homozygous for 56%, with nine private alleles shared by both individuals (Table S4B).

### 3.3. Galveston Canids Carry Coyote mitochondrial DNA Haplotypes

We found the two classically supported canid mtDNA clades: (1) Eurasian-evolved gray wolves and domestic dogs, and (2) North American canids (coyotes, eastern wolves, and red wolves; posterior probability = 0.98) (Figure 1D). Both GI canids carried coyote haplotypes derived from the Great Plains (GI-1: haplotype la77; accession JN982588; 32) and Texas (GI-2: la143; accession FM209386; 17), and both clearly grouped with North American canids, although nodal support within clades was generally low (Figure 1D and Figure S4; posterior probability <0.5), especially for coyotes which show very little phylogenetic structuring across their range [39] (Figure 1D and Figure S4).

## 4. Discussion

We rediscovered red wolf ghost alleles present in the American southeast nearly 40 years after they were extinct in the region. Through interbreeding with coyotes, this endangered genetic variation has persisted and could represent a reservoir of previously lost red wolf ancestry. This unprecedented discovery opens new avenues for innovative conservation efforts, including the reintroduction of red wolf ghost alleles to the current captive and experimental populations. Consequently, these admixed individuals are of great conservation value, yet the ESA currently lacks any explicit policy providing protection for admixed individuals that serve as reservoirs for extinct genetic variation. An ‘intercross policy’ was introduced in 1996 to assist prioritizing protection efforts but was never fully adopted [40]. Several commentaries have encouraged an updated implementation of the ESA and Species Status Assessments, especially as admixed genomes are increasingly being described and viewed as a source of potentially beneficial genetic variation in the face of rapid climate change (e.g., [41]). Although red wolves represent one of the greatest species recovery stories in ESA history, debates regarding historical and ongoing interbreeding with coyotes highlight the ESA’s short-comings associated with admixed individuals and the difficulty in setting management objectives given our evolving understanding of admixed genomes across wild populations [42].

Our analyses revealed a surprising amount of allele sharing with the captive breeding population of red wolves. This shared variation could be the consequence of two potential scenarios: (1) Surviving ancestral polymorphisms from the shared common ancestor of coyotes and red wolves that have drifted to a high frequency in the captive breeding red wolf population and in a small portion of Gulf Coast coyotes; or (2) coyotes in the Gulf Coast region are a reservoir of red wolf ghost alleles that have persisted into the 21st century. Neither of these potential explanations require adherence to a specific species concept. For instance, incomplete lineage sorting from a shared common ancestor could occur whether red wolves are a subspecies of the gray wolf, conspecific with Eastern wolves, or an independent lineage with a possible ancient hybrid origin [43] (Figure S5). Similarly, interbreeding with the ancestral red wolf population would have resulted in the introgression of red wolf alleles and associated phenotypes into Gulf Coast coyotes under each species concept. Our findings of admixture and composition of private alleles are most consistent with the second scenario, where the Galveston Island canids are admixed coyotes carrying red wolf ghost alleles. Further, Galveston Island is found within the historic red wolf range from where the original founders for the captive and reintroduced populations were captured in the 1970s (Figure S6). This island population likely experienced reduced gene flow with southeastern coyotes. In further support that coyotes of the American Gulf Coast likely serve as a ghost allele reservoir of red wolf ancestry, we also identified two coyotes with red wolf admixture from Louisiana’s Gulf Coast, a second geographic region in which trapping efforts were conducted to build a captive red wolf population [16]. These findings provide substantial support that ancestral red wolf genetic variation persists as ghost alleles in the regional coyotes of the southeastern United States.

While our primary objective was to determine the extent of red wolf allele sharing among the Galveston Island canids, our discovery warrants further genetic surveys of coyote populations in Louisiana and Texas to establish the level and extent to which remnant red wolf alleles are found exclusively in admixed coyotes. There are potentially admixed coyotes in the region that exhibit higher levels of red wolf ancestry, as exemplified by the two Louisiana coyotes that also exhibited partial assignment to the red wolf cluster. Broadly, admixture levels in southeastern coyotes could be impacted by variation in habitat, hunting, and dispersal barriers across the region. Given gunshot mortality is known to increase coyote–wolf hybridization [11,12], there may be a need to regulate coyote hunting until we know more about the frequency of endangered wolf genetics in the American Gulf coast. With genetic surveys in place, conservation efforts then face the opportunity to consider the role of remnant genetic variation in the future of the red wolf. The NCEP of red wolves is a listable entity under the ESA in need of proactive conservation [43]. However, in the age of an extinction crisis, innovative mechanisms to preserve and utilize adaptive potential are in great demand. Today, every federally recognized red wolf individual is a descendant from 14 founders, of which only 12 are genetically represented. These founders were removed from a single geographic location in the 1970s and vastly underrepresent the original genomic diversity present in southeastern wolves [5]. Our discovery of red wolf ghost alleles in southeastern coyotes demonstrates the ability to uncover ancestral variation and establish a new component of biodiversity conservation. A minority of conservation priorities have considered a ‘de-introgression’ strategy in which admixed individuals are bred in a specific design to recover the extinct genotype [44]. For instance, after identifying wild canids with red wolf ghost alleles, a breeding program could be established to prioritize individuals representing rare red wolf ancestry with the goal of recovering lost genomic variation, similar to Reference [45].

While de-introgression may prove useful to recover extinct red wolf ancestry from admixed individuals, a new paradigm has been proposed to more broadly re-evaluate the role of admixed genomes [42,46]. Red wolves face anthropogenically-mediated hybridization, but introgression is also likely a natural process in the evolution of *Canis* lineages. As an important evolutionary process, introgression could protect adaptive potential and maintain processes that sustain ecosystems. Incorporating admixed entities into conservation policy and, here, red wolf restoration may be the next step in broader biodiversity conservation. Another pivotal step in red wolf restoration is the identification of a new reintroduction site for a wild population of red wolves. Our discovery of red wolf ghost alleles indicates there are geographic regions that can harbor endangered genetic variation and may guide future efforts for red wolf reintroduction. The foundation upon which that effort will be built rests exclusively on describing large-scale geographic patterns of red wolf ghost alleles in the American southeast.

## Figures and Tables

**Table 1:** Diversity statistics for each reference group of *Canis* in North America. Summary statistics for each *Canis* reference population derived from 7047 genome-wide polymorphic SNPs. (Abbreviations: *n*: sample size; *H*_O_: observed heterozygosity; H_E_: expected heterozygosity; A_R_: allelic richness; PA_R_: private allelic richness; *N*_PA_: number of private alleles).

Group	Sampling Location *	*n*	*H*_O_	H_E_	A_R_ *	*N*_PA_	PA_R_ **
Coyote	Southeastern USA	29	0.085	0.101	1.52	2,686	0.28
Gray wolf	Yellowstone National Park	10	0.072	0.076	1.27	368	0.10
Eastern wolf	Algonquin Provincial Park	10	0.079	0.087	1.36	332	0.11
Red wolf (captive)	Point Defiance Zoo and Aquarium	11	0.051	0.061	1.17	191	0.04

* For details regarding which U.S. states are in the sampling region, please see Table S1. ** Allelic richness and private allelic richness are reported for minimum sample size (g) of 18, the maximum obtainable g for eastern wolves and gray wolves given the sample size and tolerance threshold.

**Table 2:** Private allele sharing between reference groups and each Galveston Island, TX canid. Summary statistics for GI-1 and GI-2 were calculated over 6859 and 6391 genome-wide SNPs, respectively, reflecting the number of loci with nonmissing data for each individual. (Abbreviations: *N*_PA_: number of private alleles; percentage; *S*_PA_: shared private alleles; *S*_Par_: shared private allelic richness).

Reference Group	GI-1				GI-2			
	*N*_PA_	*S*_PA_ (Count)	*S*_PA_ (%)	*S*_PAr_	*N*_PA_	*S*_PA_ (count)	*S*_PA_ (%)	*S*_PAr_
Coyote	2632	184	6.99%	0.0102	2439	138	5.66%	0.0093
Gray wolf	362	12	3.31%	0.0035	335	10	2.99%	0.0036
Eastern wolf	329	12	3.65%	0.0045	303	8	2.64%	0.0039
Red wolf	188	21	11.17%	0.0059	171	14	8.19%	0.0063

## Supplementary Materials

The following are available online at http://www.mdpi.com/2073-4425/9/12/618/s1, Figure S1: (A) Principal component analysis (PCA) of all reference coyotes and red wolves as well as the two GI canids. (B) Cross-validation (CV) error per number of inferred clusters (*K*) in the ADMIXTURE analysis. (C) Posterior probability assignments of each GI canid implemented in STRUCTURE, Figure S2: Nested analysis of population structure in ADMIXTURE including only reference coyotes, red wolves, GI-1 and GI-2.,Figure S3: Genomic location of shared private alleles with the red wolf reference group in GI-1 (A) and GI-2 (B) calculated over 8167 and 7609 linked genome-wide SNPs, respectively, Figure S4: Phylogenetic tree estimating the relationships among canid mitochondrial control region sequences., Figure S5. An outline of the three different hypothetical species scenarios for the evolutionary relationships among North American canids., Figure S6: Reprint of the historic range map of red wolves (gray shading of map) and the location of last remnant population (red shading in the inset map),, Table S1: Sample information, species, region of collection, and provenance for each sample, Table S2: Pairwise comparisons of expected heterozygosity (H_E_) and their associated adjusted *p*-values from the Wilcoxon rank sum test across 7047 genome-wide polymorphic SNPs., Table S3: Sample information including Genbank accession number, species, region collected, sample age, and citations, for all for sequences used in gene trees assessing the relationships among canid mitochondrial control region sequences., Table S4: (A) Summary of shared private alleles with red wolves in either GI canid. (B) Summary of private alleles (i.e., absent from all reference groups) found in either GI canid.
