# Supplementary material for: Rediscovery of Red Wolf Ghost Alleles in a Canid Population Along the American Gulf Coast
Source: Genes (Basel). 2018 Dec 10;9(12):618. doi: 10.3390/genes9120618 (PMC6315914; doi:10.3390/genes9120618)
Supplement: Supplementary file 1 [file genes-09-00618-s001.zip › genes-381665-supplementary.docx]

**Supplemental Material**

**Figure S1.** **A)** Principal component analysis (PCA) of all reference coyotes and red wolves as well as the two GI canids**. B)** Cross-Validation (CV) error per number of inferred clusters (K) in the ADMIXTURE analysis. **C)** Posterior probability assignments of each GI canid implemented in STRUCTURE.


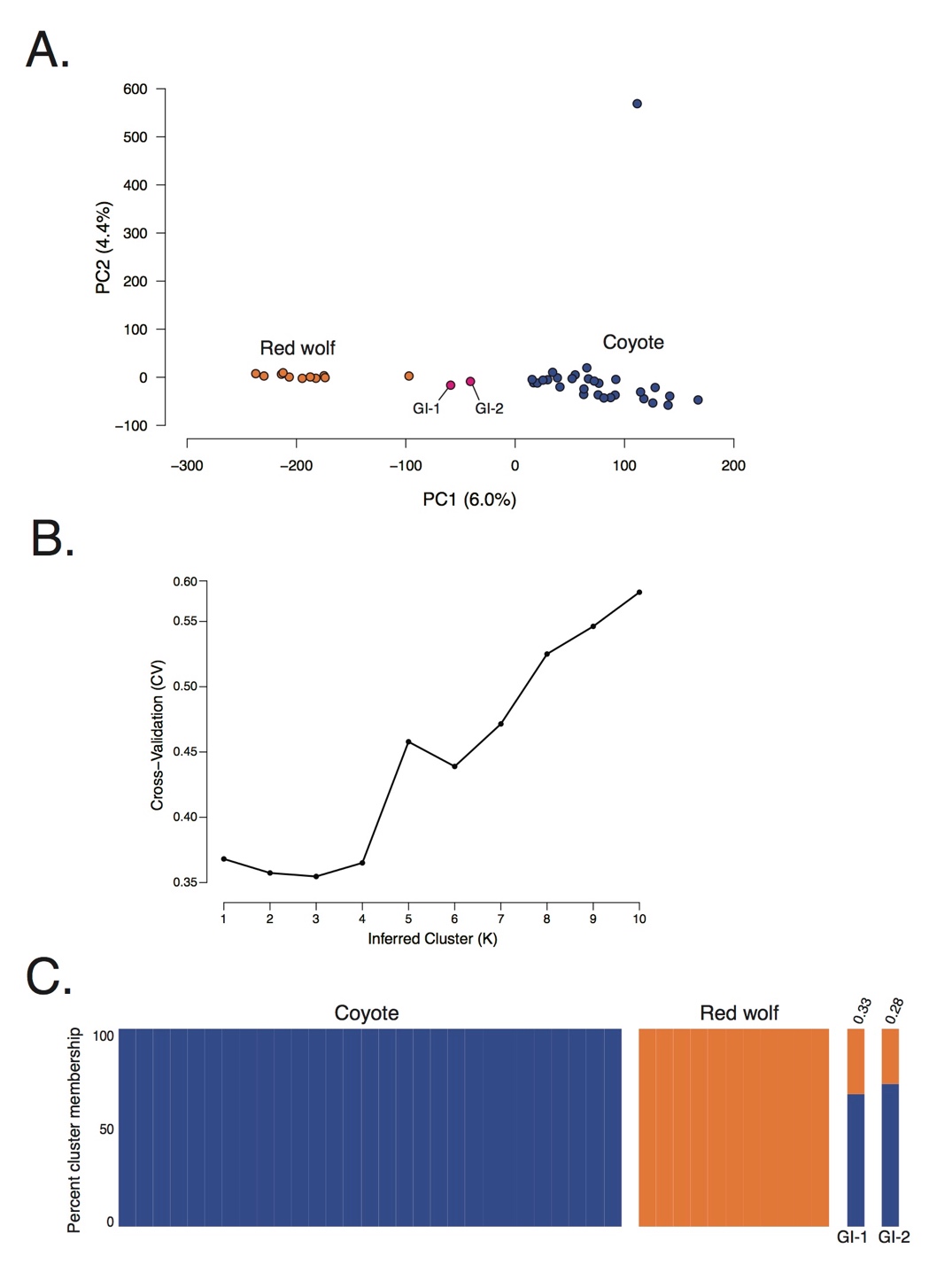


**Figure S2.** Nested analysis of population structure in ADMIXTURE including only including only reference coyotes, red wolves, GI-1 and GI-2. **A)** Cross-Validation (CV) error per number of inferred clusters (*K*) and a **B)** nested genetic structure analysis at *K*=2 (upper) and *K*=3 (lower). The southeast includes Alabama and Louisiana; the historical range includes Texas and Oklahoma.


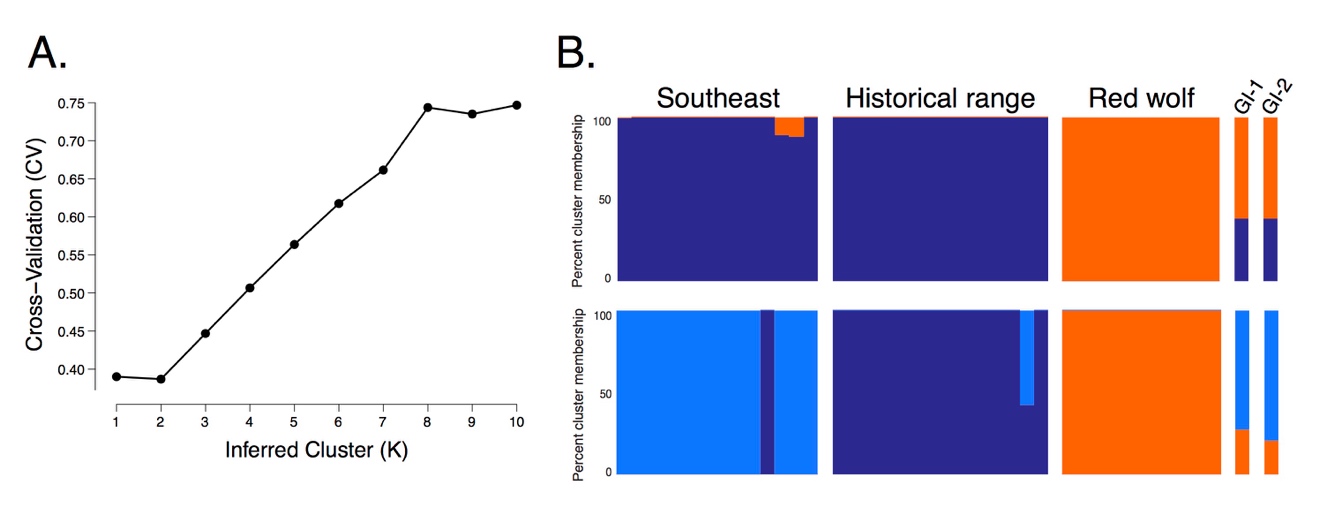


**Figure S3.** Genomic location of shared private alleles with the red wolf reference group in GI-1 **A)** and GI-2 **B)** calculated over 8,167 and 7,609 linked genome wide SNPs, respectively.


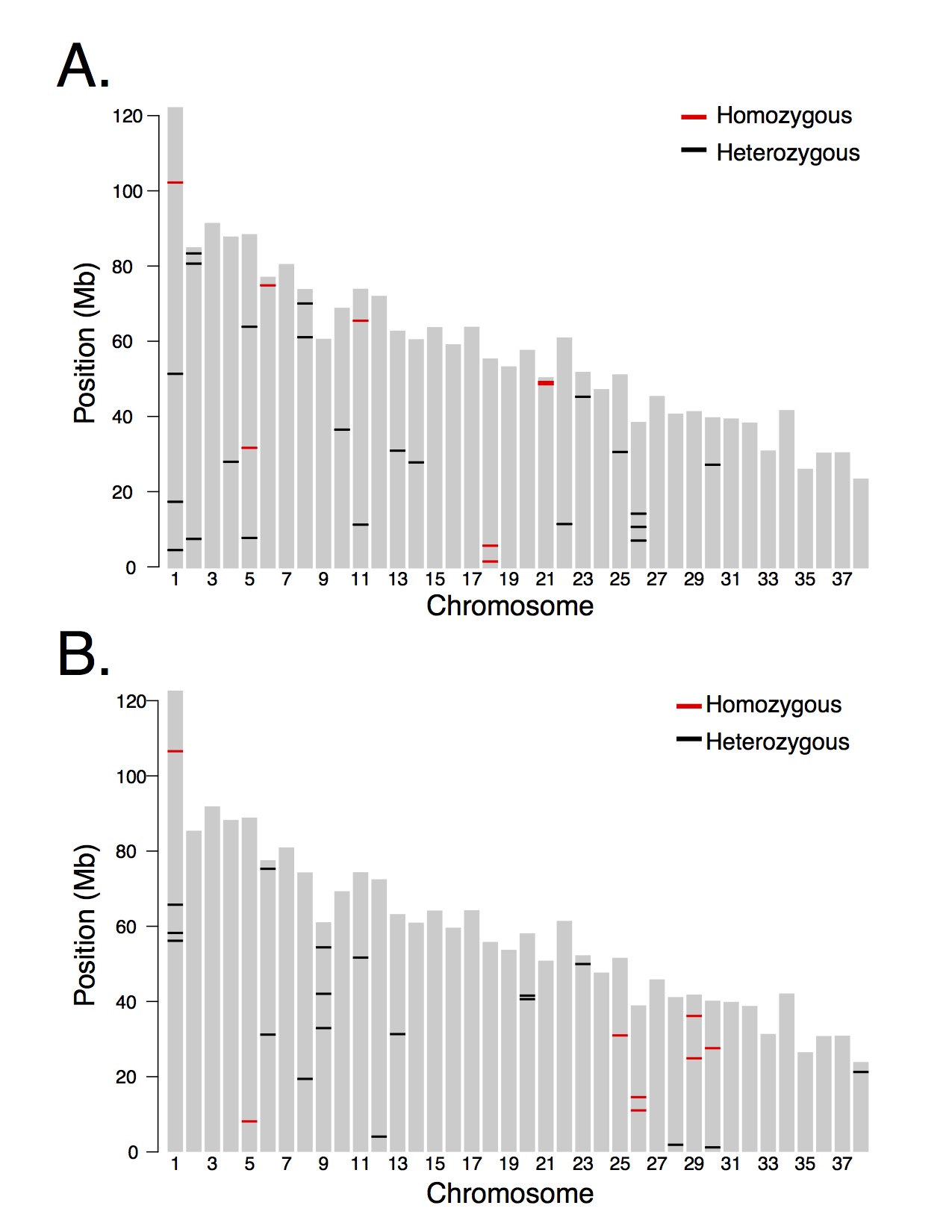


**Figure S4.** Phylogenetic tree estimating the relationships among canid mitochondrial control region sequences. Bayesian posterior probabilities above 0.90 are listed above the branches. Each color represents a different species: gray wolf (*C. lupus*), dog (*C. lupus familiaris*), coyote (*C. latrans*), eastern wolf (*C. lycaon*), red wolf (*C. rufus*), ancient canid (red wolf or coyote), and the Galveston Island canids. Tip names indicate the Genbank accession number assigned to each sequence. The gene tree is based on sequence lengths of 234 nucleotides and was estimated with a Bayesian framework; taxonomic designation of the Eastern wolf is based on assigned clade and sample location, not necessarily field identification.


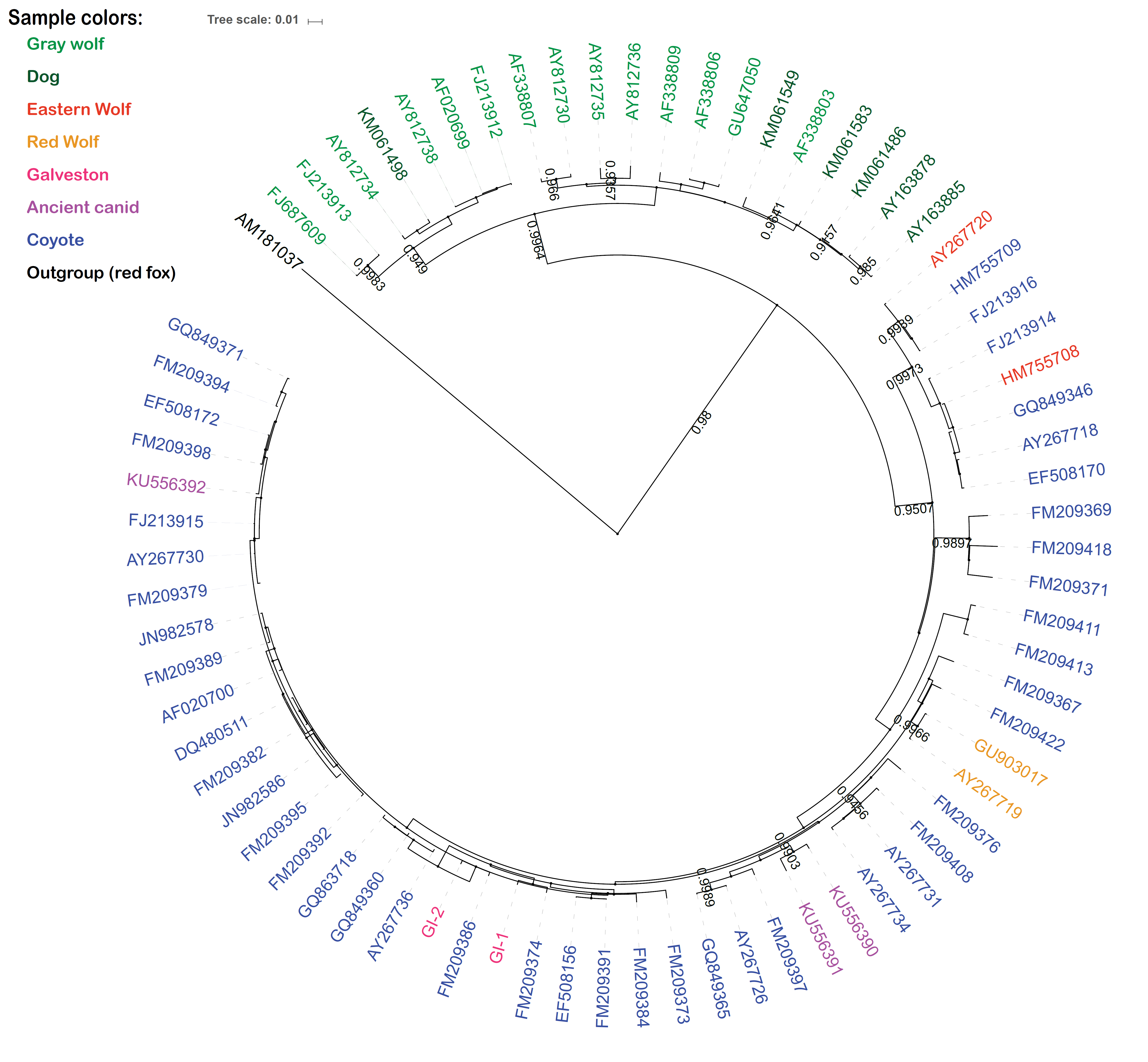


**Figure S5.** An outline of the three different hypothetical species scenarios for the evolutionary relationships among North American canids. Importantly, under each species outcome, the red wolf remains a legally listable entity under the Endangered Species Act. Adapted from [42].

**Figure S6**. Reprint of the historic range map of red wolves (gray shading of map) and the location of last remnant population (red shading in the inset map). Adapted from: Riley, G. A., & R. T. McBride. 1972. A survey of the red wolf (*Canis rufus*). U.S. Dept. Int. Spec. Sci. Rept. Wildl. No. 162. 15 p. See also: <https://www.fws.gov/southeast/wildlife/mammal/red-wolf/#range-section> for an overview of alternative historic red wolf range maps.


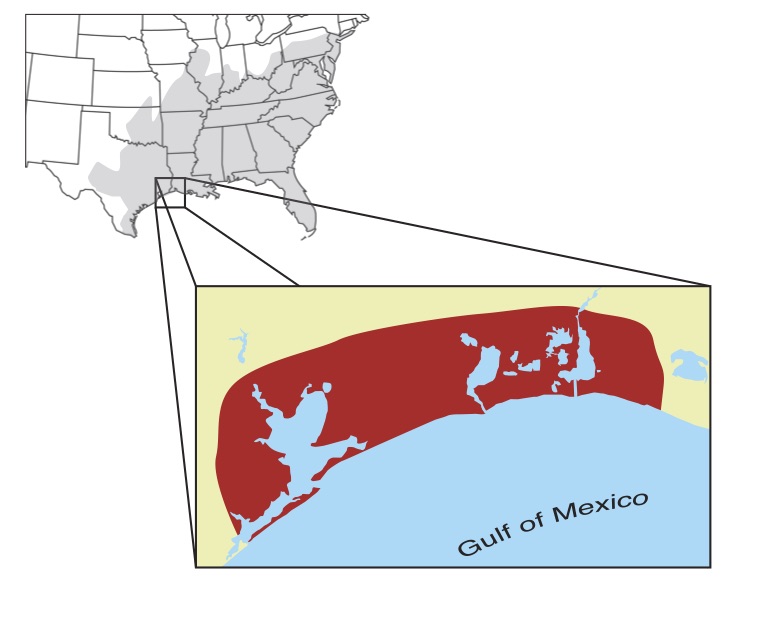


**Table S1.** Sample information, species, region of collection, and provenance for each sample.

See excel file: TableS1.xlsx

**Table S2.** Pairwise comparisons of expected heterozygosity (H_E_) and their associated adjusted *P*-values from the Wilcoxon rank sum test across 7,047 genome-wide polymorphic SNPs. For more details, see Table 1. (Abbreviations: n, sample size)

|  |  |  | *P*-value for each pairwise comparison | | |
| --- | --- | --- | --- | --- | --- |
| Group | n | H_E_ | Gray wolf | Eastern wolf | Red wolf |
| Coyote | 29 | 0.101 | 2.0x10^-16^ | 2.0x10^-16^ | 2.0x10^-16^ |
| Gray wolf | 10 | 0.076 |  | 2.0x10^-16^ | 1.1x10^-9^ |
| Eastern wolf | 10 | 0.087 |  |  | 2.0x10^-16^ |
| Red wolf | 11 | 0.061 |  |  |  |

**Table S3.** Sample information including Genbank accession number, species, region collected, sample age, and citations, for all for sequences used in gene trees assessing the relationships among canid mitochondrial control region sequences. Bolded, italicized samples are represented in the reduced tree in text made for easier viewing. The full dataset was used in tree construction.

See excel file: TableS3.xlsx

**Table S4.** **A)** Summary of shared private alleles with red wolves in either GI canid. **B)** Summary of private alleles (i.e. absent from all reference groups) found in either GI canid.

**A)**

| Chromosome | Position (bp) | Genomic context | GI-1 Genotype | GI-2 Genotype |
| --- | --- | --- | --- | --- |
| chr01 | 17,732,301 | Intron | Heterozygous | Missing |
| chr01 | 102,633,286 | Exon | Homozygous | Wild Type |
| chr02 | 7,849,790 | Intergenic | Heterozygous | Wild Type |
| chr02 | 81,065,628 | Intergenic | Heterozygous | Wild Type |
| chr02 | 83,781,834 | Intron | Heterozygous | Wild Type |
| chr04 | 28,362,431 | Intergenic | Heterozygous | Wild Type |
| chr05 | 32,069,379 | Intergenic | Homozygous | Missing |
| chr05 | 64,278,740 | Promoter | Heterozygous | Missing |
| chr06 | 75,287,848 | Intergenic | Homozygous | Heterozygous |
| chr08 | 19,414,126 | Intergenic | Wild Type | Heterozygous |
| chr08 | 61,491,893 | Intergenic | Heterozygous | Wild Type |
| chr08 | 70,464,831 | Intergenic | Heterozygous | Wild Type |
| chr09 | 32,923,622 | Intron | Wild Type | Heterozygous |
| chr09 | 54,400,294 | Intergenic | Wild Type | Heterozygous |
| chr10 | 36,913,557 | Intergenic | Heterozygous | Wild Type |
| chr11 | 51,669,090 | Intron | Wild Type | Heterozygous |
| chr13 | 31,321,750 | Intergenic | Heterozygous | Heterozygous |
| chr18 | 1,846,578 | Exon | Homozygous | Wild Type |
| chr20 | 40,620,197 | Intergenic | Wild Type | Heterozygous |
| chr21 | 48,991,281 | Intergenic | Homozygous | Wild Type |
| chr22 | 11,804,844 | Intergenic | Heterozygous | Wild Type |
| chr23 | 45,655,764 | Intergenic | Heterozygous | Wild Type |
| chr25 | 30,978,579 | Intron | Heterozygous | Homozygous |
| chr26 | 7,413,320 | Intergenic | Heterozygous | Wild Type |
| chr26 | 11,036,335 | Intergenic | Heterozygous | Homozygous |
| chr28 | 1,894,226 | Intron | Wild Type | Heterozygous |
| chr29 | 36,144,569 | Intergenic | Wild Type | Homozygous |
| chr30 | 1,208,506 | Intron | Wild Type | Heterozygous |
| chr30 | 27,583,020 | Intergenic | Heterozygous | Homozygous |
| chr38 | 21,253,893 | Exon | Wild Type | Heterozygous |

**Table S4 (*continued*).**

**B)**

| Chromosome | Position (bp) | Genomic context | GI-1 Genotype | GI-2 Genotype |
| --- | --- | --- | --- | --- |
| chr2 | 17,295,908 | Intergenic | Wild Type | Homozygous |
| chr2 | 51,857,680 | Intergenic | Heterozygous | Heterozygous |
| chr2 | 78,496,480 | Intergenic | Wild Type | Homozygous |
| chr4 | 55,790,578 | Intergenic | Wild Type | Homozygous |
| chr5 | 469,034 | Intergenic | Heterozygous | Homozygous |
| chr5 | 25,626,376 | Intron | Homozygous | Wild Type |
| chr7 | 4,494,634 | Intergenic | Heterozygous | Heterozygous |
| chr8 | 71,035,539 | Intergenic | Homozygous | Missing |
| chr10 | 3,130,326 | Intergenic | Heterozygous | Heterozygous |
| chr10 | 38,016,421 | Intergenic | Wild Type | Homozygous |
| chr12 | 58,523,836 | Intron | Homozygous | Heterozygous |
| chr14 | 35,633,927 | Intron | Heterozygous | Heterozygous |
| chr16 | 10,571,931 | Intergenic | Homozygous | Heterozygous |
| chr18 | 52,849,477 | Intron | Heterozygous | Homozygous |
| chr23 | 18,744,824 | Intergenic | Homozygous | Wild Type |
| chr27 | 9,245,99 | Exon | Heterozygous | Heterozygous |
| chr29 | 7,288,666 | Intergenic | Homozygous | Wild Type |
| chr29 | 25,210,498 | Intergenic | Wild Type | Homozygous |
| chr30 | 4,352,137 | Intergenic | Homozygous | Wild Type |
| chr30 | 35,778,062 | Intergenic | Wild Type | Homozygous |
| chr34 | 10,260,789 | Intergenic | Wild Type | Homozygous |
